# Potential excess of vaginal examinations during the management of labor: frequency and associated factors in 13 Peruvian hospitals

**DOI:** 10.1186/s12978-019-0811-9

**Published:** 2019-10-10

**Authors:** Jessica Hanae Zafra-Tanaka, Renee Montesinos-Segura, Pamela D. Flores-Gonzales, Alvaro Taype-Rondan

**Affiliations:** 1 CRONICAS Center of Excellence in Chronic Diseases, Universidad Peruana Cayetano Heredia, Lima, Peru; 2 Escuela Profesional de Medicina Humana, Universidad Nacional de San Antonio Abad del Cusco, Cusco, Peru; 3 Facultad de Medicina Humana, Universidad Nacional del Altiplano, Puno, Peru; 4 Unidad de Investigación para la Generación y Síntesis de Evidencias en Salud, Universidad San Ignacio de Loyola, Av. la Fontana 550, La Molina, Lima, Peru

**Keywords:** Vaginal examinations, Obstetric labor, Childbirth

## Abstract

**Background:**

A high number of vaginal examinations (VEs) may lead to a higher risk of infections, as well as discomfort/dissatisfaction with intrapartum care.

**Objective:**

To determine the frequency of potential excess of vaginal examinations (PEVE) during the management of labor and identify its associated factors, in Peruvian hospitals.

**Methods:**

Secondary analysis of the data collected in the *DisrespEct and abuse during ChIlDbirth in pEru (DECIDE)* study, held between April and May 2016. In this study, women hospitalized in Peruvian hospitals right after giving birth were surveyed by trained personnel. PEVE, the main outcome, was considered as five or more vaginal examinations (VEs) performed during the management of labor. Poisson regression models with robust variance were performed to calculate crude and adjusted prevalence ratios (cPR and aPR) as well as their 95% confidence intervals (95% CI).

**Results:**

One thousand four hundred twenty registries of 13 hospitals from 8 Peruvian cities were evaluated. The number of women studied at each hospital ranged between 100 and 129. The median age was 26 years (interquartile rank: 22–31). The median number of VEs was 3 (interquartile rank: 2–5). The proportion of women who underwent PEVE was 33.9%, this ranged from 0.9 to 69.9% at the studied hospitals. The frequency of PEVE was higher in women who attended > 2 obstetric psychoprophylaxis sessions, compared to those who attended ≤ 2 sessions (aPR: 1.78 95% CI: 1.01–3.12); and among women who gave birth between 18:00 h and 23:59 h, compared to those who did it between 7:00 and 17:59 h (aPR: 1.28 95% CI: 1.04–1.57).

**Conclusion:**

Around one in three women underwent a PEVE, although this frequency varied widely across the evaluated hospitals. Women with more psychoprophylaxis sessions, and who gave birth between 18:00 h and 23:59 h, had a higher PEVE frequency. Future studies should assess in depth the causes and consequences of this high frequency.

## Plain English summary

A high number of vaginal examinations (VEs) may lead to a higher risk of infections, as well as discomfort/dissatisfaction with intrapartum care. We aimed to evaluate how frequent the potential excess of vaginal examinations (PEVE) was during management of labor and identify its associated factors in Peruvian hospitals.

We performed a secondary analysis of the data collected in the *DisrespEct and abuse during ChIlDbirth in pEru (DECIDE)* study, held between April and May 2016. This study included women hospitalized right after giving birth. For this study we defined PEVE as five or more VEs performed during the management of labor.

We included 1420 women. The proportion of women who underwent PEVE was 33.9%, varying between studied hospitals. Attending to more than two psychoprophylaxis sessions and giving birth between 18:00 h and 23:59 h were associated with PEVE.

In conclusion, the proportion of women who underwent PEVE was large and varied among the evaluated hospitals. Thus, there is a need to evaluate the application of guidelines regarding the frequency of VE conducted during labor, and the number of VEs needs to be evaluated relative to duration of labor.

## Background

Vaginal examination (VE) is a procedure used frequently during the management of labor [1, 2], especially to assess its beginning and to evaluate its progress [3, 4]. However, the use of VE during labor has not shown to be useful for improving outcomes of interest like length of labor, maternal or infant mortality and morbidity [5]. Moreover, recent studies have found that assessment of labor could be performed more accurately using ultrasound [6, 7].

Although a higher number of VE has not been clearly associated with an increased risk of infection or fever [5, 8–10], it has been associated with pain, discomfort, embarrassment [3, 11], dissatisfaction with intrapartum care [12], and posttraumatic stress syndrome [13, 14]. Therefore, according to the recommendations provided by the World Health Organization (WHO), the number of VE performed during labor should be limited to what is strictly necessary and should be performed to confirm the beginning of labor and every 4 hrs to identify prolonged labor [15].

The excessive number of VE during labor has been assessed in previous studies. However, few of them have taken place in Latin America [9, 16–18] and Peru [19]. Moreover, few studies have evaluated the associated factors to the excessive number of VE during labor [3, 4, 20], which is important to design interventions assessing subgroups of women in a higher risk of being subjected to an excessive number of VE.

Due to the importance of the subject for adequate childbirth care, and to the limited number of studies conducted in Latin America and Peru, the present study aimed to determine the potential excess of vaginal examinations (PEVE) performed during the management of labor and to identify its associated factors in Peruvian hospitals.

## Methods

### Study design

This is a secondary analysis of the *“DisrespEct and abuse during ChIlDbirth in pEru (DECIDE) study”* database [21], which was carried out between April and May 2016, and aimed to evaluate disrespect and abuse during childbirth in Peruvian hospitals.

### Study sample

The primary study collected information from 1528 puerperal women who were hospitalized in the maternity wards of 14 hospitals in nine cities of Peru: two from the coast, five from the highlands, and two from the jungle. Those who did not wish to participate in the study were not able to respond to the survey, or whose babies had died were not included in the study.

For the present study, records from one of the hospitals included in the primary study were excluded from the analysis since surveys took there did not include certain covariates of interest for the present analysis. In addition, those records of women who did not provide information on the number of VEs performed during labor and records containing inconsistent data were excluded.

### Settings

In Peru, four health systems coexist: public, social security, armed/police, and private systems [22]. Facilities included in this study belong to the public and social security health systems, which give coverage to 58.0% of the Peruvian population. Public system provides care for the informal workers, independent workers, and unemployed; whereas social security system provides health insurance for the formal workers, independent workers who pay a minimum fee, retired, and their families [23].

### Procedures

Interviewers from nine Peruvian cities were recruited, and permissions for the main study were asked in all hospitals located in each city, and 14 of these hospitals granted their authorization. Considering the interviewers’ availability of time and the permissions granted at each hospital, it was established that at least 100 women were interviewed per hospital. A random date for each hospital was chosen between April and May 2016, from which interviewers assessed all postpartum women who were hospitalized in maternity wards and who had given birth within 48 h prior to the survey. Interviewers attended to the hospital with a daily to 3 times a week frequency until reaching at least 100 respondents. Frequency varied within hospitals due to the interviewer possibilities. Women signed an informed consent form before their participation and were conducted to a private environment for the survey application. A more detailed description of the methodology of the DECIDE study has been previously published [21].

### The potential excess of vaginal examinations

The collection of VEs data was done through an interview in which the postpartum women were asked about the number of VEs performed in total during labor. VE was defined as the introduction of one or more fingers into the vagina for evaluation means.

PEVE was defined as the performance of five or more VE during labor, in accordance with the national guideline for childbirth care that applies to all health facilities in Peru, in which 4 is considered the maximum number of expected VEs in normal labor [24]. This definition is in agreement with the maximum expected number of VEs recommended by the WHO, since the average duration of labor is 12 h [2], and the WHO recommends that VE be performed every four hours [15]. This cut-off has been also used in two previous studies [3, 25].

### Other variables

In order to carry out our analysis, we considered three socio-demographic variables: age (in tertiles), educational level (without education, complete primary, complete secondary, or complete higher education), region where the hospital is located (coast, highlands, or jungle), and hospital health system (public or social security).

Current pregnancy-related variables were: number of deliveries including the recent one (1 or ≥ 2), number of prenatal controls (in tertiles), and referral from another health facility during labor (yes or no). Current delivery variables included: type of delivery (vaginal or cesarean), giving birth on the weekend (yes or no), and delivery time (between 00:00 and 06:59 h, between 07:00 and 17:59 h, or between 18:00 and 23:59 h).

The number of obstetric psychoprophylaxis sessions during pregnancy was also collected. Psychoprophylaxis can be defined as the integral, theoretical, physical, and psycho-affective preparation provided during pregnancy, with the objective of preparing women for future obstetric care [26]. This variable was categorized to compare the upper tertile with the lower two (0–2 versus ≥3 psychoprophylaxis sessions).

### Data analysis

Absolute and relative frequencies were calculated for categorical variables. Medians, interquartile ranks (IQR), means, and standard deviations, were calculated for quantitative variables.

In order to determine the factors associated with PEVE, crude and adjusted prevalence ratios (cPR and aPR) and their 95% confidence intervals (95% CI) were calculated using crude and adjusted Poisson regression models with robust variance. The adjusted model included the following variables: age, educational level, region, health system, number of deliveries considering the recent one, number of prenatal controls, number of psychoprophylaxis sessions, referral, type of delivery, day of delivery, and time of delivery. For the adjusted model hospitals were entered as clusters. Data analysis was performed using the statistical software Stata v12 (StataCorp, TX, US).

### Ethics

Ethical approval for the baseline study, DECIDE, was obtained from the Institutional Review Boards at the *Hospital Madre-Niño San Bartolomé* (RCEI-40), in Lima, Peru. Informed consent form for the participants included the purpose of the study. Participation was anonymous and voluntary, and the confidentiality of the data was ensured.

## Results

The DECIDE study addressed 1538 women at 14 hospitals, 10 women refused to participate in the study thus 1528 (99.3%) postpartum women were included. Of these, 99 surveys of one hospital were excluded because they did not have complete variables of interest. In addition, 5 records were excluded for not having information about the number of VEs performed during labor, and other 4 records were excluded because they lacked other variables of interest. Finally, data from 1420 puerperal women (92.9% of the women surveyed) was analyzed. These women were attended in 13 hospitals of eight cities in Peru.

### Demographic description

Data from 1420 postpartum women were analyzed. Of these, 988 (69.5%) were treated at nine public hospitals. The number of observations analyzed in each hospital varied between 100 and 129 (Table 1). Median age was 26 years (IQR: 22–31). Regarding the number of deliveries, 869 (61.1%) had no previous deliveries. Regarding current delivery, 553 (38.9%) had a cesarean delivery, and 634 (49.4%) were attended between 7:00 h and 17:59 h (Table 2).

**Table 1:** Number of vaginal examinations per hospital

Hospital	Surveyed women (*N* = 1420)	Number of VEs (Median and IQR)	% of PEVE (95% CI)
Public hospitals			
Highlands hospital 1	129	5 (4 to 6)	69.0 (61.0–77.0)
Highlands hospital 2	110	5 (4 to 7)	60.9 (51.8–70.0)
Jungle hospital 1	109	4 (3 to 5)	45.0 (35.6–54.3)
Jungle hospital 2	110	4 (3 to 5)	42.7 (33.5–52.0)
Coast hospital 1	108	3 (2 to 5)	29.6 (21.0–38.2)
Highlands hospital 3	110	3 (2 to 4)	15.5 (8.7–22.2)
Highlands hospital 4	100	3 (2 to 4)	8.0 (2.7–13.3)
Highlands hospital 5	100	2 (2 to 3)	1.0 (0.0–3.0)
Coast hospital 2	110	2 (2 to 3)	0.9 (0.0–2.7)
Social Security hospitals			
Coast hospital 3	108	6 (4 to 10)	63.9 (54.8–72.9)
Jungle hospital 3	110	4 (3 to 5)	46.4 (37.0–55.7)
Highlands hospital 6	106	4 (2 to 6)	46.2 (36.7–55.7)
Coast hospital 4	110	2 (2 to 3)	1.8 (0.0–4.3)

VEs: Vaginal examinations.

PEVE: Potential excess of vaginal examinations (≥ 5 VEs).

**Table 2:** Characteristics of the evaluated population (N = 1420)

Characteristics	Total N (%)
Hospital characteristics	
Region	
Coast	436 (30.7)
Highlands	655 (46.1)
Jungle	329 (23.2)
Health system	
Public	986 (69.4)
Social security	434 (30.6)
Socio-demographic characteristics	
Age	
13 to 23 years	482 (33.9)
24 to 29 years	505 (35.6)
30 to 45 years	433 (30.5)
Educational level	
Without education	364 (25.6)
Complete primary	120 (8.5)
Complete secondary	462 (32.5)
Complete higher	474 (33.4)
Pregnancy and delivery characteristics	
Number of deliveries (considering the recent one)	
1	552 (38.9)
≥ 2	868 (61.1)
Number of prenatal controls	
< 6	386 (27.2)
6 to 9	895 (63.0)
≥ 10	139 (09.8)
Number of psychoprophylaxis sessions	
0 to 2	1010 (71.2)
≥ 3	409 (28.8)
Referred from another health facility	521 (36.7)
Cesarean delivery	552 (38.9)
Giving birth during the weekend	350 (24.7)
Time of delivery	
Dawn (00:00–06:59 h)	361 (28.2)
Afternoon (7:00–17:59 h)	633 (49.4)
Night (18:00–23:59 h)	288 (22.5)

### The potential excess of vaginal examinations and its associated factors

**Fig. 1:**
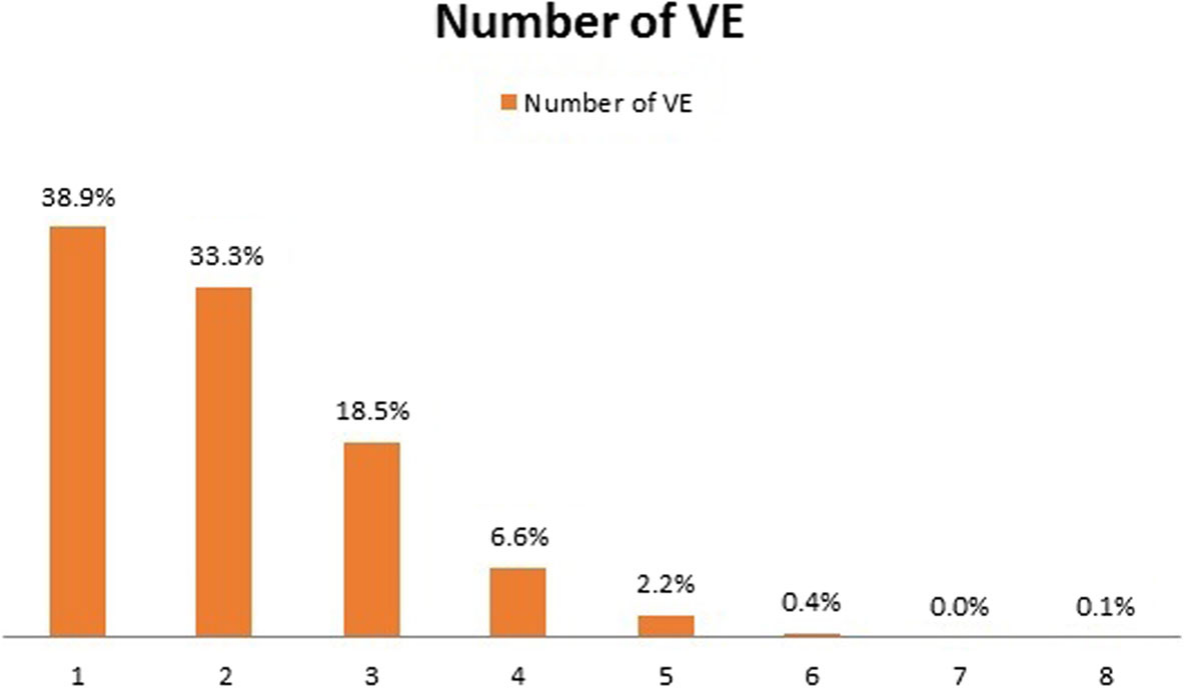
Frequency of vaginal examinations performed to Peruvian women

The median number of VEs was 3 (IQR: 2 to 5). The hospital with the lowest median presented 2 VE (IQR: 2 to 3) and the highest 6 VEs (IQR: 4 to 10). PEVE, defined as ≥5 VEs during the management of labor, was reported by 33.9% of all women, ranging from 0.9 to 68.9% at different hospitals (Table 1  and Fig. 1).

**Fig. 2:**
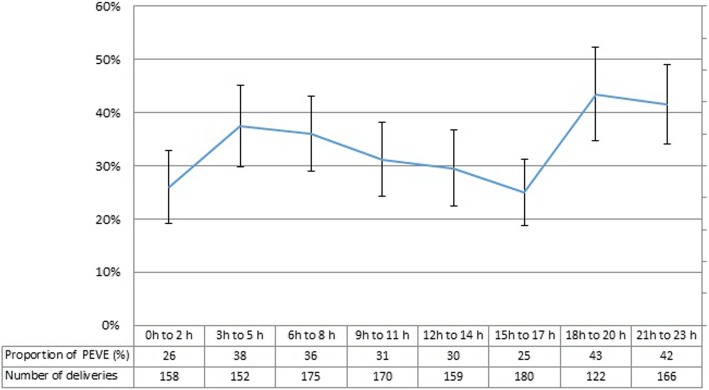
Proportion of women who underwent potential excess of vaginal examinations by time of birth, and 95% CI

Postpartum women who attended to ≥3 psychoprophylaxis sessions had a higher frequency of PEVE (aPR: 1.78, 95% CI: 1.01–3.12). In addition, those patients whose delivery took place between 18:00 h and 23:59 h had a higher frequency of PEVE compared to those whose delivery was attended between 7:00 h and 17: 59 h (aPR: 1.28, 95% CI: 1.04–1.57) (Table 3 and Fig. 2).

**Table 3:** Associated factors to potential excess of vaginal examinations in Peruvian women

Characteristics	No PEVE N (%)	PEVE N (%)	Crude model PR (95% CI)	Adjusted model * PR (95% CI)
Age				
13 to 23 years	336 (69.7)	146 (30.3)	Ref.	Ref.
24 to 29 years	332 (65.7)	173 (34.3)	1.13 (0.88–1.45)	1.18 (0.93–1.50)
30 to 45 years	270 (62.4)	163 (37.6)	1.24 (0.92–1.68)	1.32 (0.93–1.88)
Educational level				
Without education	201 (55.2)	163 (44.8)	Ref.	Ref.
Complete primary	96 (80.0)	24 (20.0)	0.45 (0.14–1.41)	0.67 (0.30–1.49)
Complete secondary	314 (68.0)	148 (32.0)	0.72 (0.40–1.27)	0.90 (0.52–1.55)
Complete higher	327 (69.0)	147 (31.0)	0.69 (0.36–1.33)	0.76 (0.40–1.45)
Region				
Coast	332 (76.1)	104 (23.9)	Ref.	Ref.
Highlands	424 (64.7)	231 (35.3)	1.48 (0.42–5.26)	1.41 (0.69–2.87)
Jungle	182 (55.3)	147 (44.7)	1.87 (0.62–5.62)	1.53 (0.73–3.23)
Health system				
Public	675 (68.5)	311 (31.5)	Ref.	Ref.
Social security	263 (60.6)	171 (39.4)	1.25 (0.56–2.79)	1.33 (0.86–2.06)
Number of deliveries considering the recent one				
1	355 (64.3)	197 (35.7)	Ref.	Ref.
≥ 2	583 (67.2)	285 (32.8)	0.92 (0.73–1.16)	0.85 (0.69–1.04)
Number of prenatal controls				
< 6	222 (57.5)	164 (42.5)	Ref.	Ref.
6–9	645 (72.1)	250 (27.9)	0.66 (0.40–1.09)	0.77 (0.58–1.02)
≥ 10	71 (51.1)	68 (48.9)	1.15 (0.63–2.11)	1.31 (0.82–2.08)
Number of psychoprophylaxis sessions				
0 a 2	742 (73.5)	268 (26.5)	Ref.	Ref.
≥ 3	195 (47.7)	214 (52.3)	**1.97 (1.10–3.52)**	**1.78 (1.01–3.12)**
Referred				
No	625 (69.5)	274 (30.5)	Ref.	Ref.
Yes	313 (60.1)	208 (39.9)	1.31 (0.73–2.34)	1.55 (0.94–2.55)
Type of delivery				
Vaginal	568 (65.4)	300 (34.6)	Ref.	Ref.
cesarian	370 (67.0)	182 (33.0)	0.95 (0.67–1.36)	0.99 (0.74–1.32)
Day of delivery				
Not weekend	715 (66.8)	355 (33.2)	Ref.	Ref.
Weekend	223 (63.7)	127 (36.3)	1.09 (0.93–1.28)	1.07 (0.95–1.21)
Time of delivery				
Dawn (00:00–06:59 h)	243 (67.3)	118 (32.7)	1.10 (0.79–1.53)	0.97 (0.76–1.24)
Afternoon (7:00–17:59 h)	445 (70.3)	188 (29.7)	Ref.	Ref.
Night (18:00–23:59 h)	166 (57.6)	122 (42.4)	1.43 (0.99–2.06)	**1.28 (1.04–1.57)**

* Adjusted for all the variables showed in the table

PEVE: Potential excess of vaginal examinations (≥ 5 VEs).

## Discussion

### Summary of results

We evaluated 13 hospitals in eight Peruvian cities. The proportion of postpartum women who underwent PEVE was 34%. However, it varied between 1 and 70% across the studied hospitals. PEVE was associated with attending to a greater number of obstetric psychoprophylaxis sessions and the time of delivery, with a higher frequency of PEVE for those puerperal women who gave birth between 18:00 h and 23:59 h compared to those whose delivery took place between 7:00 h and 17:59 h.

### The proportion of women subjected to PEVE

The mean number of VEs performed during labor in our study was 3.96 ± 2.5. The hospital with the lowest mean of VEs had 2.1 ± 0.8 and the one with the highest mean presented 6.8 ± 4.4. Other studies also showed great variability in the number of VEs, with 2.9 ± 1.5 in a hospital in Scotland [4], 4.0 ± 1.7 in a hospital in Peru [19], 4.2 ± 2.1 in a Palestinian hospital [3], 4.5 ± 3.7 in two hospitals in Mexico [16], and 5.6 ± 3.5 in two hospitals in Panama [17].

The proportion of women who underwent PEVE in our study was also highly variable among the hospitals, and the pooled proportion was slightly lower than the 36% [3] and 44% [25] reported in two different hospitals in Palestine with the same cut-off point.

Although the Peruvian and the WHO guidelines establish that VEs should be performed every 4 hrs to evaluate the progress of labor [15, 24], we found a great variability among Peruvian hospitals. We propose four features that could explain this variability. First, it is possible that health personnel in each hospital tend to mainly follow indications of professionals with more experience, considered as experts in the area [27, 28]. Thus, regarding VEs, such opinions may be different from the guideline recommendations. Second, there are some procedures that are usually performed accompanied by VE such as intrapartum analgesia, artificial rupture of membranes, or placement of fetal electrodes [20, 29, 30]. Thus, it is possible that these procedures are overused in certain hospitals. Third, in hospitals with higher amount and diversity of health personnel performing the assessment of the progress of labor (including gynecologists, midwives, residents, interns, medical students, and obstetrics students), each health personnel could register his findings on different documents and carry out parallel VE [31]. Accordingly, the number of health professionals involved in labor management has found to increase the number of VEs [3]. Fourth, the staff in training could be performing unnecessary VE just for training reasons, or necessary VE that later will be corroborated by more experienced personnel.

These features should be studied in depth to prevent an excessive number of VEs. In addition, it is necessary to generate local evidence regarding number or frequency of VEs associated to discomfort, embarrassment, or dissatisfaction with intrapartum care in Peruvian women, in order to correctly generate cut-off points for VE.

### VE and psychoprophylaxis sessions

Women who had assisted to ≥3 sessions of psychoprophylaxis had a higher frequency of PEVE. One of the goals of these sessions is for pregnant women to recognize the beginning of labor and the timing in which they should go to the health center [32]. Therefore, the higher frequency of PEVE associated with a greater number of psychoprophylaxis sessions could be explained by the fact that women who have been trained in recognizing the onset of labor attend earlier to health facilities and are exposed more time to health care.

### VE and time of delivery

A higher frequency of PEVE was observed in women whose delivery took place between 18:00 h and 23:59 h. Conversely, in a study conducted in a hospital in Palestine, no differences were found in the frequency of PEVE according to the time of delivery [3]. Our results can be attributed to the shift that usually takes place at 18 h or 20 h. The incoming team has usually been working all morning, so it is understandable that they try to reduce their workload by reducing the number of patients they have in charge. In order to achieve this, they may opt for more active management of labor which includes rupture of membranes and stimulation with oxytocin, procedures which are usually preceded by VE [33, 34]. These patients may be the ones who gave birth between 18:00 and 23:59 h.

It is also possible that women who delivered between 18 and 23:59 h were assessed in the afternoon, during which gynecologists are usually performing medical procedures and staff in training may take the opportunity to develop skills in the performance of VE, as reported in a teaching hospital in the Netherlands [35].

### Limitations

Our study had some limitations: 1) Postpartum women may have been intimidated by the hospital environment, which could have influenced their response to the number of VE. Nevertheless, in order to reduce this bias the anonymity of the survey was emphasized, and surveys were performed without the presence of health professionals in the room. 2) Recall bias for the number of VE is possible. Thus, efforts were made to survey women just few hours after their birth, assess them in a quiet environment, and give them enough time to remember the characteristics of their birth. Also, to minimize these limitations, further studies should corroborate the information collected through the revision of the hospital registries. 3) Our definition of PEVE (≥ 5 VEs) applies only if average labor lasted 12 h in the hospital. Thus, pregnant women who arrived with advanced labor and underwent VE with a frequency greater than the recommended (one every 4 h) could be falsely labeled as not PEVE, which could be underestimating the PEVE frequency found. In order to have a more accurate approximation of excess of VE, some studies have opted to calculate the expected number of VE by dividing the number of hours the in-patient was in labor in four [4, 28]. Unfortunately, we had no information about the length of labor spent in the hospital. 4) Health professionals may need to perform extra VEs in specific cases such as presence/suspicion of a dysfunctional labor pattern or abnormal fetal heart rate pattern. However, we were not able to collect these situations, so we had no clinical information to evaluate if extra VEs were or were not necessary for labor management. Nevertheless, we expect to compensate this limitation, at least partially, by using the mean of expected VEs.

Despite these limitations, our results are important as they show wide differences among different hospitals, as well as a temporal variation in the number of VE, which may reflect differences in the intensity of management of labor according to the time of birth.

## Conclusions

Around one in three women underwent PEVE (1 to 70% across hospitals) of women underwent a PEVE during the management of their labor in the studied hospitals. Those with a greater number of psychoprophylaxis sessions and those who gave birth between 18:00 h and 23:59 h had a higher frequency of PEVE. Future studies should assess in depth the causes and consequences of this high frequency.

## Data Availability

The datasets used and/or analyzed during the current study are available from the corresponding author on reasonable request.

## Abbreviations

PEVE: potential excess of vaginal examinations
VE: Vaginal examination
WHO: World Health Organization
